# Frequency and Molecular Identification of *Cryptosporidium* Species among Immunocompromised Patients Referred to Hospitals, Central Iran, 2015–16

**Published:** 2020

**Authors:** Shahrokh IZADI, Mohammad Ali MOHAGHEGH, Zahra GHAYOUR-NAJAFABADI, Mehdi AZAMI, Farzaneh MIRZAEI, Fatemeh NAMDAR, Mehdi MOHEBALI, Dhammika LESHAN WANNIGAMA, Seyed-Hossein HEJAZI

**Affiliations:** 1. Department of Medical Parasitology and Mycology, School of Medicine, Isfahan University of Medical Sciences, Isfahan, Iran; 2. Department of Laboratory Sciences, School of Paramedical Sciences, Torbat Heydariyeh University of Medical Sciences, Torbat Heydariyeh, Iran; 3. Health Sciences Research Center, Torbat Heydariyeh University of Medical Sciences, Torbat Heydariyeh, Iran; 4. Skin Diseases and Leishmaniasis Research Center, Isfahan University of Medical Sciences, Isfahan, Iran; 5. Department of Medical Parasitology and Mycology, School of Medicine, Shahid Sadoughi University of Medical Sciences, Yazd, Iran; 6. Department of Laboratory Sciences, School of Para-Medicine, Shahid Sadoughi University of Medical Sciences, Yazd, Iran; 7. Department of Medical Parasitology, School of Public Health, Tehran University of Medical Sciences, Tehran, Iran; 8. Center for Research of Endemic Parasites of Iran (CREPI), Tehran University of Medical Sciences, Tehran, Iran; 9. Department of Microbiology, Faculty of Medicine, Chulalongkorn University, King Chulalongkorn Memorial Hospital, Bangkok, Thailand; 10. Davison of Pediatrics, School of Medicine, Faculty of Health and Medical Sciences, The University of Western Australia, Nedlands, Western Australia, Australia

**Keywords:** *Cryptosporidium*, Immunocompromised patients, Genotype, Iran

## Abstract

**Background:**

The purpose of this study was to determine the prevalence and genotype of *Cryptosporidium* spp. in different groups of immunocompromised patients admitted to the referral hospitals in center of Iran during 2015–2016.

**Methods:**

This cross-sectional study was conducted on 346 immunocompromised patients (HIV^+^/AIDS, Lymphoma, Leukemia and organ transplants) in referred hospitals from central parts of Iran including Isfahan, Markazi, Yazd and Chaharmahale Bakhtiari provinces. Stool samples were analyzed for *Cryptosporidium* species, modified Ziehl–Neelsen staining techniques followed by the semi-nested PCR and DNA sequencing methods.

**Results:**

The total rate of *Cryptosporidium* spp. was 3.46% (12/346) in the patients, however, the prevalence of the parasite, was 4.6% (4/87) in HIV^+^/AIDS patients, 3.6% (6/168) in patients with blood malignancy and 2.1% (2/91) in organ transplant recipients. The SSU rRNA gene of *Cryptosporidium* spp. in all microscopic-positive samples was effectively amplified by the semi-nested PCR and DNA sequences, exposed the existence of two *Cryptosporidium* species, including *C. hominis* 91.6% (11/12) and *C. parvum* 8.3% (1/12).

**Conclusion:**

The predominance of *C. hominis* in the present study may be certifies the importance of anthroponotic transmission of cryptosporidiosis in center of Iran.

## Introduction

Nowadays, the number of immunocompromised patients is increasing. They are those with congenital anomalies, HIV/AIDS, organ transplant recipients, cancer patients as well as an enormous population that take immunosuppressive drugs for other disorders (1). Numerous opportunistic infections happen in immunocompromised patients, as a result of impairment of immune system (2) among them *Cryptosporidium* spp. has been known as an important opportunistic enteric parasite in these patients (3). The organism has a wide-range host that comprises humans and domestic animals all over the world. In developing countries, *Cryptosporidium* infections take place generally in children younger than 5 yr and in industrialized societies, epidemic cryptosporidiosis can occur in adults via foodborne or waterborne outbreaks (4). Humans acquire *Cryptosporidium* infections through several routes, such as direct contact with infected persons and animals and/or the ingestion of contaminated food and water (5). This microorganism is an opportunistic parasite causing severe diarrhea, fever, anorexia, dehydration and eventually weakness in immunocompromised patients. The main problem in proper identification of *Cryptosporidium* spp. is to distinguish oocysts from other small particles in fecal and environmental specimens such as yeasts, molds, algae, and plant debris (6). Currently, 26 species are recognized as valid on the basis of morphological, biological and molecular data. Of the nearly 20 *Cryptosporidium* species and genotypes reported in humans, *Cryptosporidium hominis* and *Cryptosporidium parvum* are responsible for the majority of infections (7). However, handful of studies on the epidemiology and classification of *Cryptosporidium* species in central Iran are available.

Therefore, the present study aimed to genotypes isolated from immunocompromised patients, in four provinces including Chaharmahale Bakhtiari, Isfahan, Markazi and Yazd, central Iran

## Methods

### Ethical consideration

This study was approved by Ethics Committee of Isfahan University of Medical Sciences (Isfahan, Iran). In addition, written informed consent was obtained from all the participants before stool sampling. (Ethical approval number: IR.MUI.REC.1396.3.305).

### Sampling

**Fig. 1:**
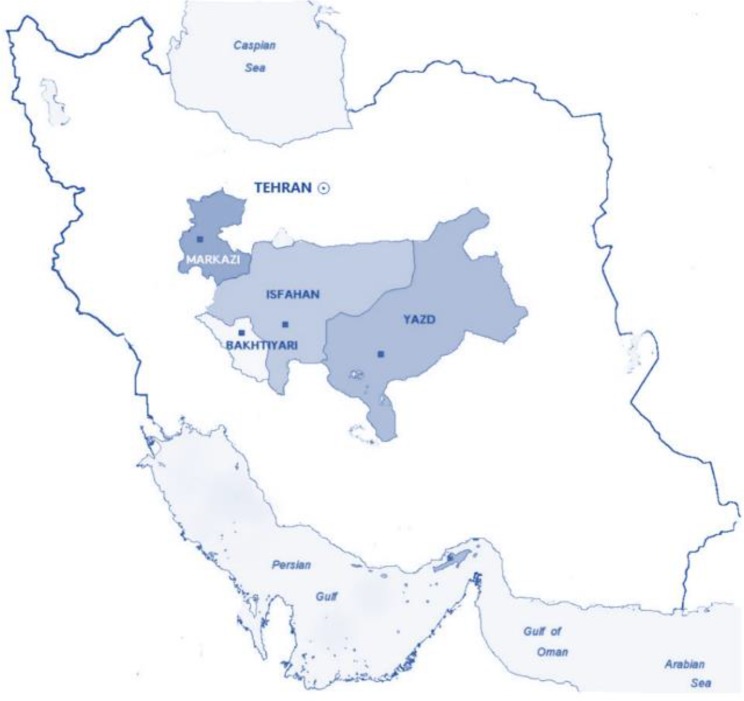
Studied provinces in central Iran including Isfahan, Yazd, Markazi, and Chaharmahal and Bakhtiari

Overall, 346 fecal samples were obtained from different groups of immunocompromised ambulatory and hospitalized patients (>15 yr old) including HIV/AIDS, lymphoma, leukemia and organ transplants as of four provinces including Isfahan, Markazi, Yazd and Chaharmahale Bakhtiari located in the central parts of Iran during 2015–2016 (Fig. 1). Of these, 26 patients conferred with diarrhea and 320 did not have diarrhea. All samples were examined by microscopic observation of direct smears applying normal saline and formalin ether concentration techniques and a slide were prepared for each sample, stained with the modified Ziehl-Neelsen acid-fast technique to finding *Cryptosporidium* as described previously (8). The samples diagnosed as *Cryptosporidium* positive by microscopic examination were stored in 2.5% potassium dichromate at 4 °C before to DNA extraction.

### DNA extraction

To maximize DNA extraction for the molecular processing, an initial step of 10 freeze-thaw cycles (freezing in liquid nitrogen for 2 min and heating at 95 °C for 4 min in a water bath) were used to rupture the oocysts for all positive samples and then DNA extraction was carried out using the QIAamp ^®^ Fast DNA Stool Mini Kit 50 (Qiagen, Germany) according to the company’s instructions. Finally, based on the kit instruction, DNA was purified and stored at −20 °C until it was used for PCR analyses.

### Semi-nested PCR

A two-step semi-nested PCR protocol for the 18S rRNA gene was performed. The outer forward primer 5′-GGA AGG GTT GTA TTT ATT AGA TAA AG-3′ and common reverse primer Cr 550 5′-TGA AGG AGT AAG GAA CAA CCT CC-3′ were described previously (9). They amplified a ∼835-bp fragment applying the following procedure: 95 °C for 5 min followed by 25 cycles of 94 °C for 40 sec, 53 °C for 30 sec, and 72 °C for 45 sec, and the last extension of 72 °C for 5 min. The second round was performed using the forward primer Cr250 5′-GGA ATG AGT KRA GTA TAA ACC CC-3′ and the reverse primer Cr 550 5′-TGA AGG AGT AAG GAA CAA CCT CC-3′ that could amplify a region with ∼530 bp. The second PCR reaction used 95 °C for 5 min, followed by 35 cycles of 94 °C for 35 sec, 55 °C for 30 sec, and 72 °C for 40 sec, and over a final extension step at 72 °C for 5 min. Amplification done in final volume of 25 μL, containing 12.5 μL of 2X PCR master mix (Ampliqon, Denmark), 10.5 pM of each primer, and 2 μL of DNA using Biorad thermocycler. A sample excluding DNA as negative and a known *C. parvum* as positive controls were added in each set of PCR. The PCR product was transferred to a 1.5% agarose gel, electrophoresed for 1 h. The gel was stained with safe green and the products were visualized under a UV transilluminator (Uvitec, UK).

### DNA sequencing

PCR products were rubbed out from the agarose gel, purified and sequenced directly with the secondary PCR primers using an ABI 3730 sequencer (Bioneer, South Korea), and results were compared with sequences previously pledged in the GenBank database, using National Center for Biotechnology Information (NCBI-BLAST) software.

### Statistical analysis

The results were analyzed by Mann–Whitney, chi-square and Fisher exact tests by statistical software SPSS ver. 16 (Chicago, IL, USA) with a *P*-value of <0.05.

## Results

**Fig. 2:**
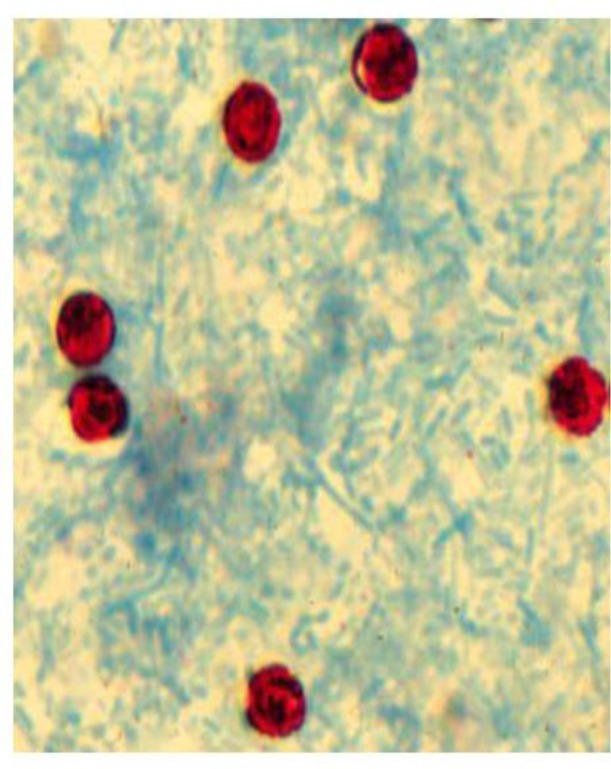
*Cryptosporidium* oocysts in the stool sample of an immunocompromised patient (×1000)

Stool samples from 346 individuals consisted of 87 (25.1%) patients with HIV+/AIDS, 168 (48.5%) patients with malignancy (Lymphoma and Leukemia), and 91 (26.3%) patients with transplant recipient were submitted to the parasitology laboratory of Isfahan University of Medical Sciences, Isfahan, Iran to find *Cryptosporidium* (Fig. 2). Numbers and percentages of people diagnosed with cryptosporidiosis in the studied provinces in Iran are shown in Table 1.

**Table 1:** The provincial frequency of *Cryptosporidium* species among immunocompromised Patients, central Iran

***Province***	***Tested Cases n (%)***	***Infected Cases n (%)***	***Cryptosporidium Species***
Isfahan	204	8(3.9)	*C. hominis*
Yazd	41	2 (4.8)	*C. hominis*
Markazi	60	0 (0.0)	-
Chaharmahal and Bakhtiari	41	2 (4.8)	*C. hominis, C. parvum*

High prevalence of HIV/AIDS patients is reported from Chaharmahale Bakhtiari Province (31.7%) and in case of blood malignancy, Yazd and Markazi provinces have the higher percentage of patients with hematologic malignancies (63.4% and 63.3% respectively) and finally, we found the highest rate of organ transplantation in Isfahan province (31.8%). The total rate of *Cryptosporidium* infection was 3.46% (12 /346) in the patients however the prevalence of the parasite was 4.6% (4/ 87) in HIV/AIDS patients, 3.6% (6/168) in patients with blood malignancy and 2.1% (2/91) in organ transplant recipients by modified Ziehl-Neelsen technique.

We found a statistically significant difference in material status (*P*=0.005) and type of stool (*P*=0.001) among the immunocompromised patients. A higher infection rate was observed in patients more 60 yr of age. There was no significant correlation between the prevalence rate of *Cryptosporidium* infection and other study variables (Table 2).

**Table 2:** Sociodemographic characteristics of immunocompromised patients, according to the presence or absence of *Cryptosporidium* species, central Iran

***Variable***		***Infected (%)***	***Non-infected (%)***	***Total***	**P*-value***
Sex	Female	4 (2.4)	162(97.6)	166	0.301
	Male	8(4.4)	172(95.6)	180	
Age group(yr)	≤ 45	4(3.4)	113(96.6)	117	0.76
	46–59	4(3.0)	131(97.0)	135	
	≥60	4(4.3)	90(95.7)	94	
Education	Illiterate	1 (1.8)	56(98.2)	57	0.53
	Elementary school	8(6.2)	121(93.8)	129	
	Secondary school or higher	3(1.9)	157(98.1)	160	
Residence	Urban	10(3.5)	274(96.5)	284	0.91
	Rural	2(3.2)	60(96.8)	62	
Occupation	Employee	2(3.1)	62(96.9)	64	0.82
	Retired	2(8.0)	23(92.0)	25	
	Self-employer	4(3.7)	103(96.3)	107	
	House keeper	3(2.6)	113(97.4)	116	
	Unemployed	1(2.9)	33(97.1)	34	
Marital status	Single	0(0.0)	78(100)	78	0.005
	Married	12(5.3)	213(94.7)	225	
	Divorced	0(0.0)	43(100)	43	
Type of Stool	Normal	7 (2.2)	313(97.8)	320	0.001
	Diarrhea	5(19.2)	21(80.8)	26	

**Fig. 3:**
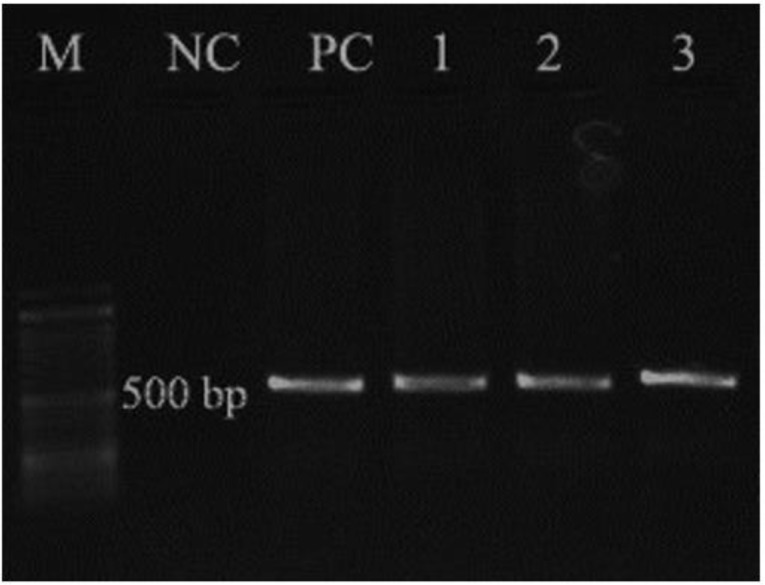
PCR of *Cryptosporidium* isolates from Iranian immunocompromised patients based on SSU rRNA gene. Lanes 1–3 *Cryptosporidium* isolates, NC: Negative control, PC: Positive control, M: DNA size marker (100 bp)

The SSU rRNA gene of *Cryptosporidium* in all 12 microscopy-positive samples was effectively amplified by the semi-nested PCR (Fig. 3).

**Fig. 4:**
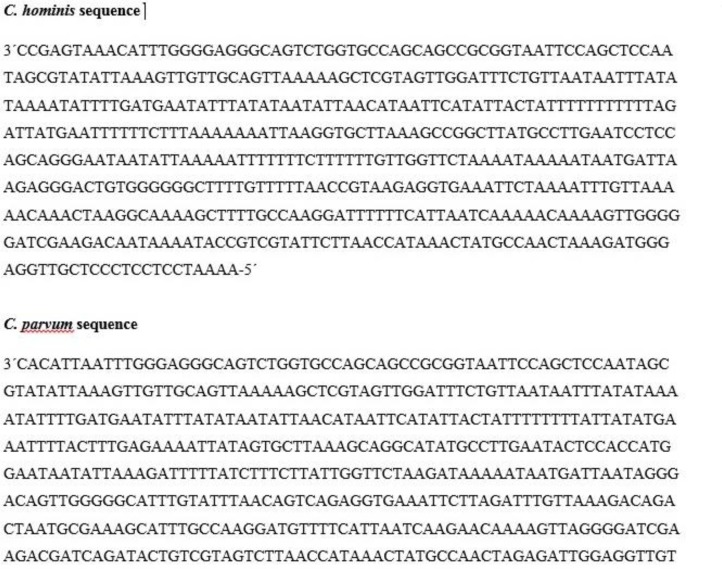
DNA sequences related to *C. hominis* and *C. parvum*

For species and genotype identification, the PCR positive products of the 18s rRNA gene of 12/346 specimens from immunocompromised patients were sequenced. Of these human specimens, 1 had 99% similarity with the *C. parvum* (GenBank accession no. AF159111); 12 had 98% similarity with the *C. hominis* (GenBank accession no. KJo19854). Fig. 4 shows the sequences of *Cryptosporidium* parasites isolated from the studied patients.

All of the positive samples from Isfahan and Yazd provinces (8 and 2 cases respectively) were *C. hominis* and one case (of 2) from Chaharmahale Bakhtiari was typed as *C. parvum* and another one was *C. hominis*. We did not find any *Cryptosporidium* in Markazi province neither with Ziehl-Neelsen nor by molecular techniques.

## Discussion

As a result of increasing the rate of immunodeficiency syndromes, cases with congenital/acquired immunodeficiency disorders, persons who suffering from malignancy undergoing chemotherapy, organ transplant recipients and patients with Human Immunodeficiency Virus (HIV), we have to pay more attention to the intestinal opportunistic parasites. Among the parasites that affect the gastrointestinal system, the protozoan parasite, *Cryptosporidium* is one of the most important intestinal organism that is a principal cause of digestive diseases in humans. This microorganism often causes chronic, and serious intestinal diseases leads to morbidity and mortality (10), therefore, cryptosporidiosis is a considerable public health problem across the world with a high variety of prevalence.Our results showed statistically significant differences between material status (*P*=0.005) and type of stool (*P*=0.001). These findings are similar to Mohaghegh et al (1) and Izadi et al (11). There was no correlation between the infection and age, sex etc. (Table 2) similar to recent studies (12, 13).

The prevalence of *Cryptosporidium* infection in immunocompromised patients in Iran was reported as 4.7% in Isfahan (2), 11.5% in Kashan (14), 35.9% in Mashhad (15) and 0.9% in Tehran (16). Epidemiological studies in Ethiopia, India, Egypt, Cameron, Malaysia, Indonesia, China, Australia, Turkey, Philippines, Iraq and Uganda was 13.2%, 21%, 60.2%, 19%, 12.4%, 4.7%, 8.3%, 2.3%, 4%, 1.9%, 18.9% and 25% respectively (17–28).

Our findings revealed that the overall frequency of the parasite was 3.46% (12 /346) in the patients. Many studies have been conducted on the prevalence of various intestinal parasites in Iran (14, 29–32) but few molecular studies have been done on the parasite in immunocompromised patients in center of Iran. Based on two studies in southern Iran, 5.1% and 9.5% of immunocompromised patients were infected with *Cryptosporidium* spp. respectively which is more than our findings and despite of our study, in another research the ELISA technique was used (12, 31).

This study accented that dominant species of *Cryptosporidium* was *C. parvum* (33 out of 34 patients), and only one patient was infected with *C. andersoni* (31), whereas in our study *C. parvum* and *C. hominis* were detected. The high proportion of *C. hominis* may be due to the great prevalence of anthroponotic cryptosporidiosis in this region. In Iran, isolates of *Cryptosporidium* from human and animal hosts were described and three species, *C. parvum*, *C. hominis*, and *C. meleagridis*, were identified, so that, *C. parvum* was the principal species found in humans which is not in agreement with our findings (33). The obvious prevalence of *C. parvum* in the report might be considered the result of zoonotic transmission (33). In Mazandaran Province in the north of Iran, in the 64 HIV+/AIDS patients, 9.4% were infected with *Cryptosporidium*. In this study, researchers have used regular techniques including formalin-ether concentration and modified Ziehl Neelsen staining, while we used the above conventional methods followed by molecular procedures. However, compared to the mentioned report, our finding revealed a less rate of *Cryptosporidium* spp. in center of Iran (34).

This difference may be due to the climate variation between the north and the central regions of Iran and also, the group studied in the study was only those with AIDS, but our study included AIDS, blood malignancies and organ transplantation. In a study on HIV/AIDS patients in Tehran, the capital of Iran, HIV-positive patients were studied and 21 subjects were found to be infected with *C. hominis* (71%) or *C. parvum* (29%). These results are similar to our study, so our findings showed the most prevalent *Cryptosporidium* species was *C. hominis*, seen in 91.6% of the study patients (35). Based on another report from Tehran, during a study conducted on children, in the 794 collected samples, 19 (2.4%) were positive for *Cryptosporidium* oocysts.

Sequences analysis showed that 17 (89.4%) of the positive isolates were *C. parvum* and 2 (10.5%) were *C. hominis* that the result is in contrast with our findings. The author overlooks the importance of zoonotic *Cryptosporidium* transmission in Iran (36). In Tehran Province, on humans and cattle, *C. parvum* was the dominant parasite causing cryptosporidiosis in humans in Iran (37) which is similar to results that revealed, all of the collected samples from children in the north of Iran (100%) were *C. parvum* and, emphasized the zoonotic transmission of cryptosporidiosis in this area (38). According to WHO reports, intestinal parasite infections are widely spread throughout the world so that some 3.5 billion people worldwide are affected, also in Iran, intestinal parasite infections are still a considerable public health problem (39).

## Conclusion

The importance of opportunistic parasitic infections among immunocompromised patients must not be neglected and we recommend additional, larger, Well-designed studies to determine the prevalence of these microorganisms. *C. hominis* was the most prevalent species in this survey, which this result is an insistence of anthroponotic transmission of cryptosporidiosis in center of Iran, however, such emphasis is still on conflict.
